# State of the Art in the Treatment of Systemic Vasculitides

**DOI:** 10.3389/fimmu.2014.00471

**Published:** 2014-10-13

**Authors:** Raashid Ahmed Luqmani

**Affiliations:** ^1^NDORMS, Rheumatology Department, Nuffield Orthopaedic Centre, University of Oxford, Oxford, UK

**Keywords:** vasculitis, cyclophosphamide, rituximab, ANCA, glucocorticoid, plasmapheresis, methotrexate, azathioprine

## Abstract

Anti-neutrophil cytoplasm antibodies (ANCA) are associated with small vessel vasculitides (AASV) affecting the lungs and kidneys. Structured clinical assessment using the Birmingham Vasculitis Activity Score and Vasculitis Damage Index should form the basis of a treatment plan and be used to document progress, including relapse. Severe disease with organ or life threatening manifestations needs cyclophosphamide or rituximab, plus high dose glucocorticoids, followed by lower dose steroid plus azathioprine, or methotrexate. Additional plasmapheresis is effective for very severe disease, reducing dialysis dependence from 60 to 40% in the first year, but with no effect on mortality or long-term renal function, probably due to established renal damage. In milder forms of ANCA-associated vasculitis, methotrexate, leflunomide, or mycophenolate mofetil are effective. Mortality depends on initial severity: 25% in patients with renal failure or severe lung hemorrhage; 6% for generalized non-life threatening AASV but rising to 30–40% at 5 years. Mortality from GPA is four times higher than the background population. Early deaths are due to active vasculitis and infection. Subsequent deaths are more often due to cardiovascular events, infection, and cancer. We need to improve the long-term outcome, by controlling disease activity but also preventing damage and drug toxicity. By contrast, in large vessel vasculitis where mortality is much less but morbidity potentially greater, such as giant cell arteritis (GCA) and Takayasu arteritis, therapeutic options are limited. High dose glucocorticoid results in significant toxicity in over 80%. Advances in understanding the biology of the vasculitides are improving therapies. Novel, mechanism based therapies such as rituximab in AASV, mepolizumab in eosinophilic granulomatosis with polyangiitis, and tocilizumab in GCA, but the lack of reliable biomarkers remains a challenge to progress in these chronic relapsing diseases.

## Introduction

The systemic vasculitides are a complex set of overlapping conditions whose natural history has been significantly modified by current therapies but continue to challenge patients and clinicians. We expect survival in over 90% (compared to over 90% mortality untreated) in the first year; about 70% with small vessel vasculitis survive up to 5 years, giving a mortality ratio of 2.6 (95% CI 2.2–3.1) compared to background (1, 2).

In large vessel vasculitis, mortality is low (3) but morbidity is high. In giant cell arteritis (GCA), visual loss occurs in up to 35% (4). In Takayasu arteritis, ischemic claudication of limbs and great vessels can require surgical reconstruction (5).

Current therapies minimize systemic and local inflammation and can preserve organ function. Immunosuppressive agents are combined with supportive management, which includes: compensating for organ dysfunction (e.g., treating hypertension or providing dialysis); dealing with or preventing comorbidity, which might arise from treatment (e.g., infection, steroid related osteoporosis, or cataract); worsening of pre-existing comorbidity (e.g., worsening of ischemic heart disease or obesity); or development of new comorbidity.

We need to ensure that we identify what we are actually treating so that we tailor the choice of treatment at the right dose and at right time for each individual.

## What are We Treating?

**Figure 1:**
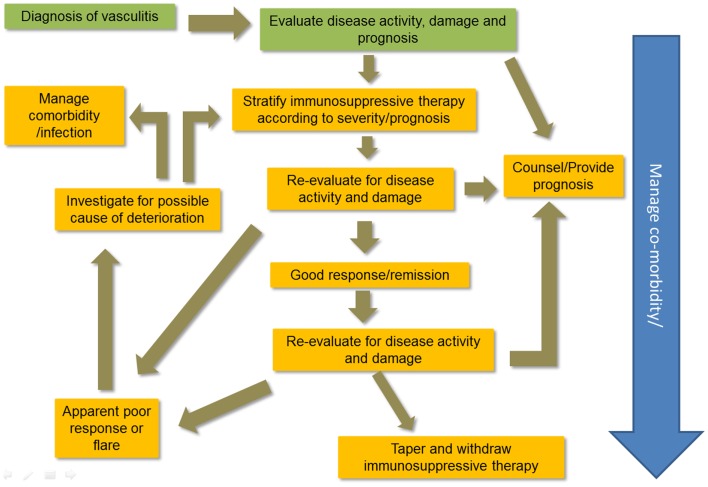
**Managing systemic vasculitis**.

Making an accurate diagnosis of the type of vasculitis is an important part of treatment choices. Figure 1 illustrates a typical plan of management for patients with vasculitis. There are no diagnostic criteria for the vasculitides; Chapel Hill Consensus Conference definitions are widely applied (6). Classification criteria for vasculitis are currently problematic (7) and research is underway to improve them (8). However, the diagnostic label is not enough. The patient’s status should include assessment of disease severity and the context in which the disease occurs in individuals. Table 1 outlines the immunosuppressive therapies used to manage vasculitis.

**Table 1:** **Immunosuppressive therapies used to treat systemic vasculitis**.

Drug	Phase of therapy	Dose	Indication/comments	Common adverse effects
**NON-BIOLOGICAL IMMUNOSUPPRESSIVE THERAPIES USED TO TREAT SYSTEMIC VASCULITIS**				
Glucocorticoids	Induction and maintenance	Varies but usually required at high initial dose (0.75–1 mg/kg/day) tapering after 4 weeks with good disease control	For GCA and Takayasu arteritis, this may be the only immunosuppression given. For most other forms of systemic vasculitis, additional immunosuppressive agents are mandatory. Increasingly, we recognize the adverse effects of glucocorticoid therapy and the aim is to minimize their use	Weight gain Hyperglycemia Mood swings Easy bruising Infection risk Cataracts Hypertension Osteoporosis Cushing’s syndrome
		Subsequent reduction of steroids is more rapid in the first 4–6 months (e.g., 5–15 mg per day reduction every 1–2 weeks), then much slower (e.g., 1 mg every 1–2 months) for large vessel vasculitis; in small and medium vessel vasculitis, because the patient is usually also managed with another immunosuppressive agent, glucocorticoid reduction protocols can be more aggressive
			In small and medium vessel multi-system disease such as GPA, MPA, EGPA, and PAN, glucocorticoid therapy remains essential to the management, except for Kawasaki disease where it is rarely used	
		Pulse high dose intravenous methylprednisolone (500–1000 mg) may be indicated for organ or life threatening manifestations, but the evidence base for its use is poor		
Cyclophosphamide	Induction	Usually given intravenously as high dose intermittent pulses of 15 mg/kg/dose on 6–10 occasions, 2–3 weeks apart. Oral pulse therapy is feasible and delivers higher level of active metabolites (due to first pass metabolism in liver to active compound)	Most forms of small vessel ANCA vasculitis, some patients with PAN and some with large vessel vasculitis require cyclophosphamide	Cytopenias Nausea and vomiting Diarrhea Hair loss Teratogenesis (avoid in pregnancy) Hemorrhagic cystitis
			Rituximab is increasingly used as an alternative for patients with ANCA vasculitis who have failed cyclophosphamide or in whom cyclophosphamide is contraindicated	
				Long-term risk of infertility and malignancy (especially bladder carcinoma) relate to cumulative dose life-time exposure especially above 35 g
		Continuous daily oral cyclophosphamide is also effective but the cumulative dose is much higher after 6 months compared to pulse therapy		
Plasmapheresis	Induction	Additional to conventional immunosuppression. No standard volume of exchange. A typical regimen would be to use between 7 and 10 exchanges (4 l each) in first 10 days of induction therapy (9). It is not clear which method of plasmapheresis (centrifugation or filtration) is superior	Evidence from one large randomized controlled trial suggests that additional plasmapheresis is renal sparing (10), but a follow up study of the same patient group suggested that the benefit did not last (11), suggesting that plasmapheresis may not be effective if used in patients with established kidney scarring	Increased risk of sepsis especially if combined with cyclophosphamide
				Potential risk of transmission of viral infection if using infected blood products
			A smaller study of 32 patients with GPA with 5-year follow up showed that plasmapheresis plus cyclophosphamide and glucocorticoids followed by ciclosporin maintenance therapy was effective in patients with a creatinine of >250 μmol/l at baseline (12)	
			Avoid plasmapheresis shortly after administration of other IV therapies (otherwise they are removed)	
Methotrexate	Induction or maintenance	15–25 mg/week oral or sc	Can be used as effective induction therapy for non-organ or non-life threatening ANCA vasculitis. It provides some additional benefit in control of GCA. Avoid use in significant renal impairment	Nausea Diarrhea Mouth ulcers Hair loss Cytopenia Liver dysfunction
Leflunomide	Induction or maintenance	10–40 mg/day	This drug is used for inflammatory arthritis but has shown benefit in patients with localized GPA	Nausea Diarrhea Mouth ulcers Hair loss Cytopenia Liver dysfunction Hypertension
Mycophenolate mofetil	Induction or maintenance	2–3 g per day	Less effective than azathioprine as a maintenance agent, nevertheless this drug has a place in management of ANCA vasculitis. As an induction agent it appears to be as effective as cyclophosphamide	Nausea Diarrhea Mouth ulcers Hair loss Cytopenia Liver dysfunction
Co-trimoxazole	Induction or maintenance	960 mg twice a day or 960 mg 3× per week if used in combination with methotrexate	This simple antibiotic has immunomodulatory effects in patients with mild GPA and has been shown to improve upper airways disease, usually in combination with steroids. At the reduced dose it can be used as prophylaxis against pneumocystis jirovecii in patients receiving other immunosuppressive agents	Beware allergy to sulfonamide Nausea Diarrhea Cytopenia (avoid full dose if combined with methotrexate)
Azathioprine	Induction Maintenance	Usually given as 2 mg/kg/day for maintenance but there is one report of using high dose intravenous pulse therapy with 1200 mg per month for 6 months in very resistant disease (13)	This is a common maintenance agent, following successful induction therapy with either cyclophosphamide or rituximab	Nausea Diarrhea Mouth ulcers Hair loss Cytopenia Liver dysfunction Non-melanoma skin tumors (advise sun protection)
Ciclosporin	Maintenance	2–4 mg/kg/day in two divided doses	Less commonly used than other agents, largely due to its nephrotoxicity	Nausea Diarrhea Gingival hyperplasia Increased facial hair Cytopenia Renal dysfunction Hypertension
Gusperimus	Relapse	0.5 mg/kg/day until neutropenia develops or for up to 21 days repeated every month for up to 6 months	Unlicensed in Europe, this immunomodulator therapy has been effective in relapsing GPA (14)	Well tolerated but limited information because of very limited use
			Reversible and predictable neutropenia
			Potential risk of sepsis
**BIOLOGICAL IMMUNOSUPPRESSIVE THERAPIES USED TO TREAT SYSTEMIC VASCULITIS**				
Intravenous immunoglobulin (IVIG)	Induction	2 g/kg single dose or divided over 5 days is typical therapy for Kawasaki disease (15). These doses are much higher than those used for immunodeficiency	Kawasaki disease is the main form of vasculitis responding to IVIG, in combination with high dose aspirin. ANCA vasculitis will respond temporarily, and this can be useful if patients are also septic, because it is an immunomodulating therapy. Check serum IgA to avoid allergic reactions in patients who are IgA deficient (because there is usually some IgA contamination). IVIG is prepared form pooled human serum, typically from thousands of donors. Viral screening of IVIG is now highly effective (previous IVIG therapy use has been associated with hepatitis C transmission)	Potential risk of transmission of viral infection if using infected blood products
				Allergic reaction in patients who are IgA deficient (due to expected levels of small amounts of IgA in the preparation)
				Headaches, flushing, fever, chills, fatigue, nausea, and diarrhea are transient reactions during infusions
Rituximab	Induction or maintenance	375 mg/m^2^ every week for 4 weeks or 1 g ×2 14 days apart are typical induction regimens. Maintenance therapy (typically 1 g single infusion) can be given every 4–6 months afterward	Increasingly used in place of cyclophosphamide as induction therapy at initial presentation or during relapse for ANCA vasculitis	Infusion reactions neutropenia hypogammaglobulinemia
				Infections (including small risk of progressive multifocal leukoencephalopathy)
				Potential for viral reactivation (e.g., hepatitis B)
				Development of other autoimmune conditions
Tocilizumab	Relapse	4 mg–8/kg per month intravenously or 162 mg sc per fortnight if <100 kg or 162 mg sc every week if ≥100 kg	Limited evidence for effectiveness in large vessel vasculitis. A randomized controlled trial in GCA is currently underway (16)	Infection risk
				Potential masking of evidence of sepsis (by down regulating production of CRP)
				Increased lipid levels
				Neutropenia
				Liver dysfunction
				Infusion reactions are rare
Mepolizumab	Resistant disease	Two different regimens are being explored: 300 mg sc every 4 weeks 750 mg iv every 4 weeks	This interleukin five inhibitor is effective in hypereosinophilic states and the iv regimen has been shown to control resistant cases of EGPA (17). A randomized controlled trial using the sc regimen is underway (http://clinicaltrials.gov/show/NCT02020889)	Limited evidence available only to date No increase in toxicity compared to placebo (e.g., fatigue, nausea)

*Patients are typically given intense induction therapy followed by maintenance. Most patients will require additional therapy to manage comorbidity and limit drug toxicity. Induction therapies can be repeated for relapse; however, it may be necessary to change the type of induction due to toxicity or poor initial response*.

The range of diseases encompassed includes small, medium, and large vessel vasculitis; small and medium vessel diseases are grouped together because the standard treatment approaches are very similar; however, they are starting to diversify as we develop more targeted agents.

For patients with a virus associated vasculitis, treatment of the virus is a prerequisite to controlling disease. Polyarteritis nodosa (PAN) related to hepatitis B (HBV-PAN), a typical form of PAN, is characterized by the absence of glomerulonephritis and the absence of anti-neutrophil cytoplasm antibodies (ANCA); relapses are rare, and never occur once viral replication has stopped and seroconversion has occurred (18). Eradication of hepatitis B is part of the management for HBV-PAN (18).Combining an anti-viral drug with plasmapheresis facilitates seroconversion and prevents the development of long-term hepatic complications of HBV. The incidence of HBV-PAN has decreased 10-fold as a result of improved blood safety and vaccination campaigns (19). In a study of 80 patients with HBV-PAN given anti-viral therapy plus immunosuppression, 5% relapsed and 30% died compared with 14.3% relapses and 48.6% deaths among 35 patients treated with immunosuppression alone. Patients who seroconverted achieved complete remission and did not relapse.

Unfortunately, the eradication of hepatitis C has been more problematic; patients with cryoglobulinaemic vasculitis may continue to require ongoing anti-viral therapy. Combination anti-viral therapy is more effective, as shown in a study of cryoglobulinaemic vasculitis; 69% of 23 cases treated with a combination of pegylated interferon alpha, ribavirin, and a protease inhibitor had achieved undetectable viral loads and a good clinical response in the majority including complete remission in 57% (20).

In small vessel vasculitis associated with ANCA, these antibodies are intricately involved in the pathogenesis (21). The role of conventional immunosuppressive agents remains important. Cyclophosphamide is the gold standard for multi-system small vessel vasculitis (22, 23); for less aggressive forms of disease, there is a potential role for leflunomide (24), methotrexate (25) or in one small series, high dose intravenous azathioprine (13). Whilst small open label studies of tumor necrosis factor (TNF) inhibition have suggested benefit (26) in disease control and improvement in abnormal endothelial dysfunction, a large randomized placebo controlled trial of etanercept, a TNF receptor protein, has shown no benefit in patients with GPA; in fact these patients had an increased risk of malignancy, which may in part have related to the inclusion of patients previously exposed to large doses of cyclophosphamide (27). Direct targeting of B cell production of antibody is an effective therapy for many but not all patients (28–30).

For patients with large vessel vasculitis such as GCA or Takayasu arteritis, the primary treatment is glucocorticoids, but as we identify disease mechanisms, we should be able to use targeted therapies, avoiding the use of high doses of steroids, which result in very significant toxicity in over 80% (31).

## Mechanism Specific vs. Global Immunosuppression

Immunosuppressive therapy results in global effects on the immune system, which can be both good and bad. Glucocorticoids produce a rapid improvement in all types of vasculitis by genomic effects on the cytosolic and more rapid non-genomic effects on the membrane bound glucocorticoid receptor (32), but these effects are short lived in small vessel vasculitis. By contrast, most patients experience significant steroid toxicity (over 80% for GCA) and this relates to the total steroid load (31). It is important to tailor the dose of steroids, often used together with an immunosuppressive agent (see Table 1), to minimize the harm, while still controlling disease.

Specific targeting of inflammatory immune mechanism in vasculitis is increasingly practical as we identify the molecular pathways that are primarily responsible for the disease. The role of complement in ANCA-associated vasculitis (33) has led to the development of targeted therapy against complement 5A (C5a), which is currently being tested in clinical trials^1^. The involvement by ANCA itself has led to development of specific B cell ablation therapy using rituximab and now belimumab^2^. As newer understanding of disease mechanisms is revealed then more targets for therapy will be identified or at least we will have better recognition of how we are affecting the underlying pathways with existing therapies.

## Can We Induce Remission?

The aim of managing the patients with vasculitis is to induce remission, which should be possible in the majority. However, this is a clinical remission not a cure and the majority of patients will relapse. We need to deliver treatment according to need without exposing patients to unnecessary risk whilst ensuring the maximum benefit. Conventional measurements of clinical remission are defined using disease activity scores, which are preferred to any current serological marker for small vessel and medium vessel vasculitis. In over 90% of patients with small vessel vasculitis, remission should be achieved by 6 months (22) using standard induction therapy. Further serial evaluation is important in order to detect and treat relapses.

By contrast, in large vessel vasculitis, the induction of remission is less easily documented. All the clinical symptoms and signs disappear rapidly with steroid treatment; by contrast, it is not so clear that we have adequately controlled disease at a sub-clinical level. Imaging is emerging as an effective technology to define disease activity. Unfortunately, it is expensive and can involve significant radiation exposure. The best imaging technique available is 18 fluorodeoxyglucose positron emission tomography with co-localized computerized tomography (FDG PET CT) to identify areas of abnormal glucose uptake. However, this involves exposure to an average of 14.4 mSv for females and 11.8 mSv for male patients (34) per scan. Nevertheless, it is important to quantify the presence of sub-clinical disease to find ways of preventing end stage ischemic complications or other vascular events such as thrombosis, dissection, and aneurysm. Non-invasive imaging protocols using magnetic resonance scans or ultrasound are being developed as ways of measuring change in disease state (35, 36).

## How Do We Measure Remission?

Serological measurements of disease activity are not reliable in systemic vasculitis (37). ANCA testing is very useful for diagnosis, but for subsequent follow up, levels can vary independently of future disease activity (38).

The Birmingham Vasculitis Activity Score (BVAS) is the most effective validated tool to document disease activity; it can be used as to define remission, response to therapy and flare (37, 39, 40). The BVAS consists of a list of typical features of active systemic vasculitis related to each body system; each item is recorded as present only if it is judged to be due to active vasculitis. This is semi-subjective because items are derived from the patient history and physical examination and cannot always be confirmed with more objective testing. However, the BVAS is valid, reliable, and widely used in clinical trials in vasculitis to define the responsiveness to various agents including cyclophosphamide, methotrexate, mycophenolate, intravenous immunoglobulin, and rituximab. It is a valuable tool for the clinicians and strongly recommended as a routine part of disease management in small and medium vessel vasculitis (40, 41). Other versions of BVAS have been validated for use in individual forms of vasculitis, such as the BVAS/Wegener’s granulomatosis, specifically for patients with GPA (42).

The Vasculitis Damage Index (VDI) is used to assess the outcome of vasculitis, by documenting the occurrence of damage as a result of having a diagnosis of vasculitis (43, 44). VDI is recommended as a cumulative measure to define the effectiveness of therapy (by limiting or preventing the accumulation of scarring). The VDI is strongly related to mortality. The presence of VDI levels of at least five points on a scale of 0–64 items (which occurs in about a third of patients (45) when measured 6 months from diagnosis) is associated with a much higher future mortality (approximately sixfold higher) than patients with less than five items of damage (46) 6 months from diagnosis.

## What Drugs Should We Use?

Each patient’s management should be based on their diagnosis and clinical state. Control of active vasculitis may be achieved using a range of therapies, depending on how rapidly and aggressively the treatment is required. The decision should be based on evidence, but interpreted for the individual to minimize harm, taking into account existing or likely co-morbidities. Some treatment protocols allow for this. For example, there are dose reductions for the dose of cyclophosphamide in older persons, those with renal impairment or with prior significant neutropenia (23).

The treatment protocol may need to be amended if unexpected changes occur in clinical status, either as a result of toxicity or if patients fail to respond to standard agents. Therapy should be withheld until inter-current infection is treated, or escalated in cases with poor initial response. Fundamental to these decisions is the regular careful clinical evaluation of patients to detect these changes. Table 1 summarizes the immunosuppressive agents commonly used as well as describing potential future therapies under investigation.

## Immunosuppressive Therapies

The main drug used is cyclophosphamide, cyclic nitrogen mustard, phosphamide ester, first used as a chemotherapeutic agent in the 1950s (47). Cyclophosphamide is a cytotoxic alkylating agent capable of killing B cells and T-cells. It is life-saving in patients with small and medium vessel vasculitis and is considered the drug of choice for multi organ disease (22).

However, the toxicity has been considerable, when used as continuous daily oral therapy for up to 2.7 years, providing over 100 g life-time exposure in some patients (48, 49), mainly due to its predicted cytotoxic effects on rapidly dividing normal cells. It can cause reversible nausea, vomiting, diarrhea, and hair loss; permanent infertility and malignancy occur with increasing cumulative doses, with an incidence of 5% at 10 years and 16% after 15 years (50). There is no absolute cut-off dose to avoid toxicity, but the recent British Society for Rheumatology guidelines for management of ANCA-associated vasculitis recommend restricting total exposure to <25 g (41). Current cyclophosphamide protocols use a much lower cumulative dose and the bladder cancer incidence is not increased (51). However, in a study of male fertility risk in patients given cyclophosphamide for sarcoma (52), a total dose of >7.5 g/m^2^ was associated with only a 10% chance of recovery of spermatogenesis compared to 70% for those given less than this dose. The use of short courses of high dose intravenous cyclophosphamide is likely to be safer than continuous daily oral therapy (53), chiefly due to the fact that the cumulative dose is typically 30–50% less.

Cyclophosphamide is effective in reducing the mortality in ANCA-associated vasculitis (22, 23). It can be given either as a pulse intravenous high dose therapy 15 mg/kg every 2–3 weeks on 6–10 occasions or as a continuous daily oral therapy at 2 mg/kg/day (54). The latter results in much higher cumulative dose of drug over 6 months period and there is evidence of equivalent of these two regimens; although the use of pulse cyclophosphamide is associated with a higher relapse rate (23). For less aggressive forms of vasculitis, there is a potential role for leflunomide (24), methotrexate (23, 25, 55) or in one small series, high dose intravenous azathioprine (13), and mycophenolate mofetil (56). These agents are usually less toxic but also less effective than cyclophosphamide.

For patients with large vessel vasculitis such as GCA or Takayasu arteritis, the primary treatment is to suppress systemic inflammation with glucocorticoid therapy in order to prevent significant vascular complications (such as loss of sight in GCA or aortic aneurysm formation or stenosis or occlusion of peripheral arteries in Takayasu arteritis. With better understanding of disease mechanisms, we might be able to use targeted therapies, perhaps even avoiding the use of steroids, which otherwise carry very significant risk of toxicity in over 80% (31).

There is some evidence for the effectiveness of TNF inhibition in small vessel vasculitis (26), but concerns about long-term toxicity (27).In Takayasu arteritis (35), studies show improvement but are limited to small numbers and there are no randomized controlled trials. This is partly due to the problem of not having adequate end points to demonstrate a potential treatment effect as well as due to the rarity of the condition (57). Direct targeting of B cells is an effective therapy for many patients; the response (64% in complete remission by 6 months) is similar to that achieved with cyclophosphamide (53% in complete remission by 6 months) for a group of 197 patients with new or relapsing ANCA-associated vasculitis; limited evidence suggests that in 102 patients with relapsing disease previously responding to cyclophosphamide, rituximab was more effective than cyclophosphamide (67 vs. 42% in complete remission respectively, *p* = 0.01) (28–30).

For any form of B cell depletion therapy, it is logical to assume that reconstitution of B cells after the end of treatment will lead to recurrence (58), although this is disputed, with at least one study demonstrating that disease relapse was independent of B cell numbers (59). Nevertheless, maintenance rituximab substantially reduces the risk of recurrence of disease from 73 to 12% after 2 years follow up in retrospective cohort data (60). Another retrospective observational study of 89 patients treated with rituximab for ANCA-associated vasculitis (60) suggests that there was additional protection against future relapse by using maintenance azathioprine, methotrexate, or mycophenolate mofetil compared to no additional immunosuppressive treatment [the hazard ratio for relapse was 0.53 (95% CI 0.29–0.97) if a maintenance drug was given].

## Discussion

The state of the art for therapy in vasculitis has improved, but remains unsatisfactory until we can completely control or cure the disease. We can prevent early mortality in multi-system vasculitis and have reduced the immediate effects of active vasculitis on organ function. However, our aim is to further improve the likelihood of survival and also the quality of life of those who survive, ensuring that we minimize disease activity and damage, drug toxicity, and impairment of quality of life.

With better understanding of the pathogenesis of vasculitis, we can target therapy against specific disease mechanisms. Examples of these are rituximab in ANCA-associated vasculitis; mepolizumab in eosinophilic granulomatosis with polyangiitis; complement 5a inhibition in ANCA vasculitis; and potentially Interleukin-6 (IL-6) inhibition with tocilizumab in large vessel vasculitis.

We are helped in our management of the disease by earlier diagnosis, so that treatment can be initiated before organ damage is established in most cases. Whilst the ANCA test is overused (61, 62), it has helped in earlier identification of patients with systemic vasculitis (63). Greater awareness of vasculitis as a cause of unexplained medical illness is leading to better case recognition. Imaging of arteries in large vessel vasculitis may become established as an early diagnostic test (64), which might change our current management approach.

Better management of comorbidity, particularly management of sepsis, control of hypertension, or management of renal failure have changed the outcome and potentially allowed more aggressive immunosuppression to be successful. However, in first 12 months after diagnosis of ANCA vasculitis, episodes of acute sepsis are now responsible for more deaths than vasculitis itself (49).

The role of glucocorticoids in vasculitis is being challenged. Whereas, previously they have been used at high doses for prolonged periods, we recognize their harm, coupled by the benefit from more specific therapy. We should see substantial reduction in toxicity in the coming decade, as we use lower doses or even steroid free regimens to control vasculitis.

Whilst the twentieth century has been dominated by use of the therapies for vasculitis designed to treat other conditions [e.g., cancer and rheumatoid arthritis (23, 65, 66)], drugs are now been designed specifically for vasculitis. We should see significant benefits for our patients, but we need to ensure that we measure their impact (for good and for harm). In the absence of reliable circulating biomarkers we need to use structured clinical assessment to document change in disease state in response to therapy. Development of effective, prognostic biomarkers for vasculitis would allow therapy to be targeted to disease mechanisms, tempered by safety assessments to prevent untoward harm.

## Conflict of Interest Statement

The author declares that the research was conducted in the absence of any commercial or financial relationships that could be construed as a potential conflict of interest.

## Footnotes

^1^http://clinicaltrials.gov/show/NCT01275287; http://www.controlled-trials.com/ISRCTN53663626

^2^http://clinicaltrials.gov/show/NCT01663623
